# Beyond volume: hospital-based healthcare technology as a predictor of mortality for cardiovascular patients in Korea

**DOI:** 10.1097/MD.0000000000003917

**Published:** 2016-06-17

**Authors:** Jae-Hyun Kim, Yunhwan Lee, Eun-Cheol Park

**Affiliations:** a Department of Preventive Medicine and Public Health, Ajou University School of Medicine; b Institute on Aging, Ajou University Medical Center; c Institute of Health Services Research, Yonsei University; d Department of Preventive Medicine, Yonsei University College of Medicine, Seoul, Republic of Korea.

**Keywords:** cardiovascular, heart, hospital, quality, technology

## Abstract

To examine whether hospital-based healthcare technology is related to 30-day postoperative mortality rates after adjusting for hospital volume of cardiovascular surgical procedures.

This study used the National Health Insurance Service–Cohort Sample Database from 2002 to 2013, which was released by the Korean National Health Insurance Service. A total of 11,109 cardiovascular surgical procedure patients were analyzed. The primary analysis was based on logistic regression models to examine our hypothesis.

After adjusting for hospital volume of cardiovascular surgical procedures as well as for all other confounders, the odds ratio (OR) of 30-day mortality in low healthcare technology hospitals was 1.567-times higher (95% confidence interval [CI] = 1.069–2.297) than in those with high healthcare technology. We also found that, overall, cardiovascular surgical patients treated in low healthcare technology hospitals, regardless of the extent of cardiovascular surgical procedures, had the highest 30-day mortality rate.

Although the results of our study provide scientific evidence for a hospital volume–mortality relationship in cardiovascular surgical patients, the independent effect of hospital-based healthcare technology is strong, resulting in a lower mortality rate. As hospital characteristics such as clinical pathways and protocols are likely to also play an important role in mortality, further research is required to explore their respective contributions.

## 1 Introduction

Over the past 3 decades, numerous studies of volume-outcome relationships have described better patient outcomes with specific surgical procedures,^[1–5]^ as hospitals where higher volumes of such procedures are performed reflect the hospital's accumulated experience, which in turn allows them to minimize medical errors. In addition, hospitals in which higher volumes of such procedures are performed may more successfully create a clinical environment that increases patient safety and provides a wider range of treatment services, which might include expertise in critical diagnostic services. Despite these observations, results of the volume-to-outcome relationship are not necessarily uniform,^[6,7]^ and many question the applicability of previous research on both volume and outcome.^[8]^

If hospitals supply a wide range of diagnostic and treatment services, it is more likely that they will be equipped with the necessary array of systems to support such care. Therefore, in order to both manage the large number of unique conditions and meet the needs of a wide range of hospital conditions, hospitals with high levels of healthcare technology will continue to be equipped with larger and more complex systems compared with those designed to provide basic care for common diagnoses. Eventually, hospitals with greater healthcare technology will likely be associated with improved health outcomes such as lower mortality rates.^[9]^

A variety of models have been proposed to measure hospital-based healthcare technology, although its effects on clinical outcomes are unclear. Berry and Feldstein models^[10]^ have been outpaced by rapid changes in clinical services and technologies, and the Veterans Health Administration model^[11]^ is limited in practicality due to its complex algorithm. To address many of the limitations in measuring hospital systems, we applied a simple and intuitive method to capture hospital-based healthcare technology based on previous novel work.^[12]^ Its measures focus on increasing the variety of conditions managed by hospitals with corresponding increases in access to specialized services and sophisticated technologies.

We therefore sought to investigate whether hospital-based healthcare technology is related to 30-day postoperative mortality rates after adjusting for hospital volume of cardiovascular surgical procedures, using current nationwide cohort data (from 2002 to 2013). In the future, identifying hospital-based healthcare technology may allow surgeons and hospitals, regardless of practice volumes, to implement changes that will improve patient outcomes throughout the healthcare system.

## 2 Methods

### 2.1 Data sources and study design

This study used the National Health Insurance Service–Cohort Sample Database from 2002 to 2013, which was released by the Korean National Health Insurance Service. Initial National Health Insurance Service–Cohort Sample Database cohort members (n = 1,025,340) were established via stratified random sampling using a systematic sampling method to generate a representative sample of the 46,605,433 Korean residents recorded in 2002. Those members were followed up in 2013. The data comprise a nationally representative random sample of 1,025,340 individuals, approximately 2.2% of the entire population in 2002.

The healthcare utilization claims include information on prescription drugs, medical procedures, and diagnostic codes based on the International Classification of Diseases, Tenth Revision (ICD-10) and healthcare costs. If a member was censored due to death or emigration, a new member was recruited among newborns of the same calendar year.

To analyze the relationship between healthcare technology and 30-day mortality among patients with cardiovascular disease, we included patients with ICD-10 codes I20–I28 as indicated in the main diagnostic records and simultaneously included those with cardiovascular-related surgical procedures such as coronary artery bypass graft and percutaneous coronary intervention.

We analyzed a unique database of representative individual samples of cardiovascular patients hospitalized to undergo surgical procedures. We linked each patient according to license number to a separate licensure hospital database that included the calendar year. Linkage between each patient and hospital allowed us to study the association of hospital-based healthcare technology with outcome in the follow-up sample. Thus, our analysis included 11,109 cardiovascular surgical patients at baseline.

This study was approved by the Institutional Review Board of Ajou University Hospital (AJIRB-SBR-EXP-16-054).

### 2.2 Study variables

#### 2.2.1 Independent variables

Hospital volume of cardiovascular surgical procedure patients per year was ranked from low to high using the SAS Rank function (model 1). We also measured hospital-based healthcare technology based on the range of diagnostic codes of cardiovascular-related diseases according to ICD-10 code over the study period. Hospital-based healthcare technology for cardiovascular diseases per year was ranked from low to high using the SAS Rank function (model 2). Thus, hospital volume of cardiovascular patients and hospital-based healthcare technology were each categorized into 3 groups: low, medium, and high. Thus, the combined effects of healthcare technology and hospital volume of cardiovascular surgical procedures were categorized into 9 groups (model 3).

### 2.3 Dependent variables

In this study, the primary endpoint was 30-day all-cause mortality after a cardiovascular-related surgical procedure.

### 2.4 Control variables

Individual level (age, sex, residential region, patient clinical complexity level [PCCL], inpatient type, diagnostic code, and type of procedure) and hospital level (hospital type, organization type, region, bed, doctor, and magnetic resonance imaging) were included as variables affecting mortality, and all covariate variables were categorical. To adjust for the clinical severity of each patient, PCCL, inpatient type, diagnostic code, and type of procedure were assessed at an individual level.

Age was divided into 5 categories: younger than 39 years, 40 to 49 years, 50 to 59 years, 60 to 69 years, and older than 70 years. Residential regions (hospital level) were categorized as metropolitan (Seoul), urban (Daejeon, Daegu, Busan, Incheon, Kwangju, or Ulsan), or rural (neither metropolitan nor urban).

### 2.5 Statistical analysis

χ^2^ tests and multivariate logistic regression analyses were used to analyze whether general characteristics, hospital-based health care technology, and hospital volume had a relationship with all-cause mortality. For all analyses, the 2-tailed criterion for significance was *P* ≤ 0.05. All analyses were conducted using the SAS statistical software package, version 9.4 (SAS Institute Inc., Cary, NC).

## 3 Results

### 3.1 Prevalence of 30-day all-cause morality

Of the 11,109 research subjects included in our study, the prevalence of 30-day mortality was 2.2% (246 participants; Table 1 ). Of the total sample, 2.3% of those who died within 30 days were at hospitals with low healthcare technology, and 2.6% were at hospitals with a low volume of cardiovascular surgical procedures (Table 1 ).

**Table 1:**
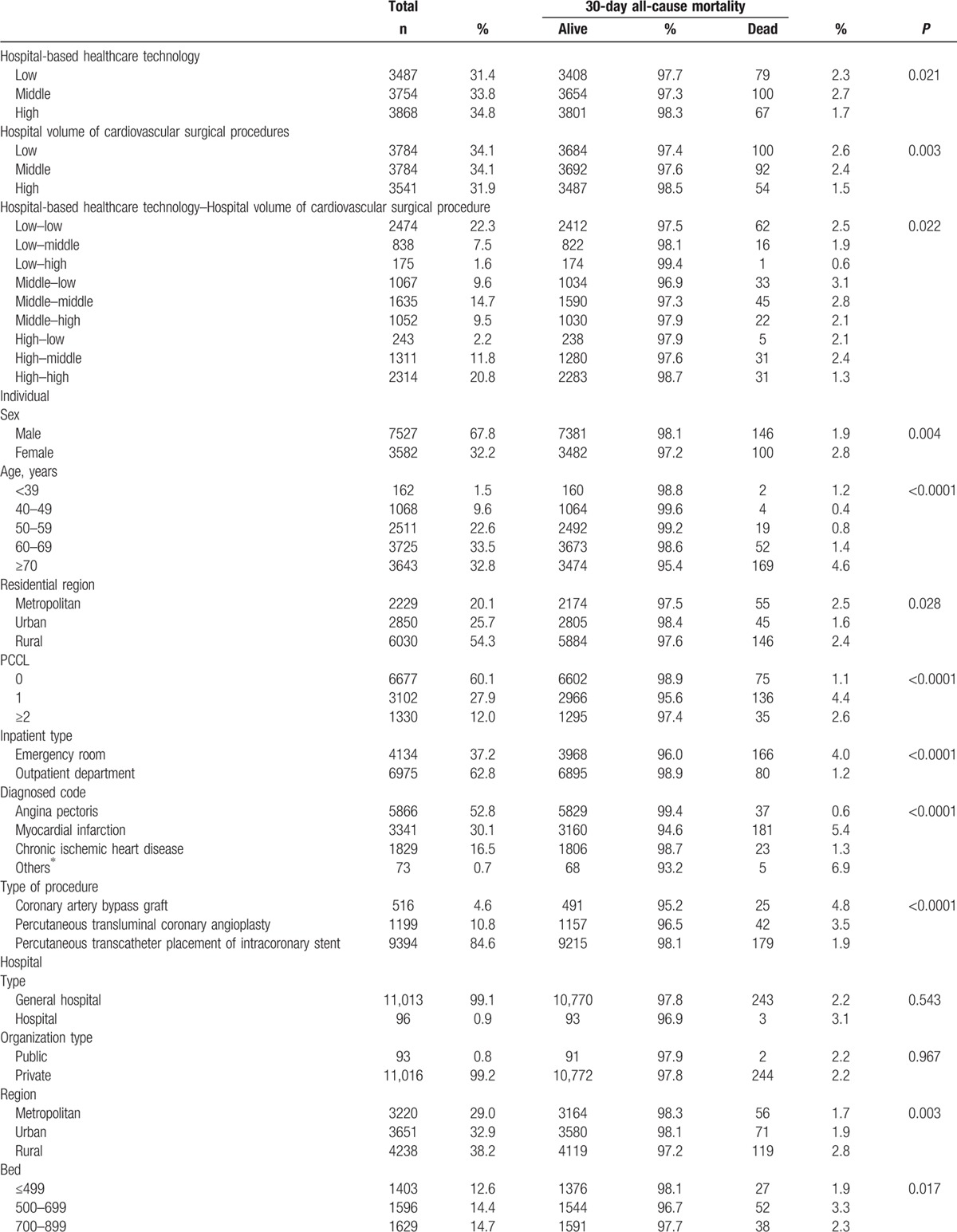
General characteristics of subjects included for analysis at baseline.

**Table 1 (Continued):**
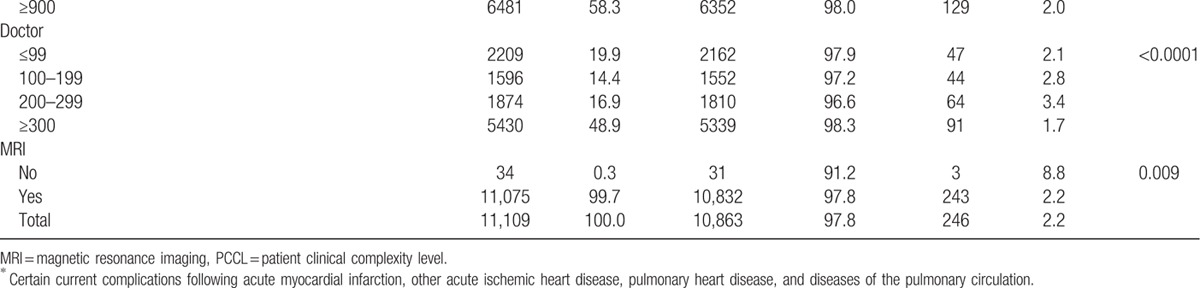
General characteristics of subjects included for analysis at baseline.

### 3.2 Association between hospital-based healthcare technology and 30-day mortality

**Figure 1:**
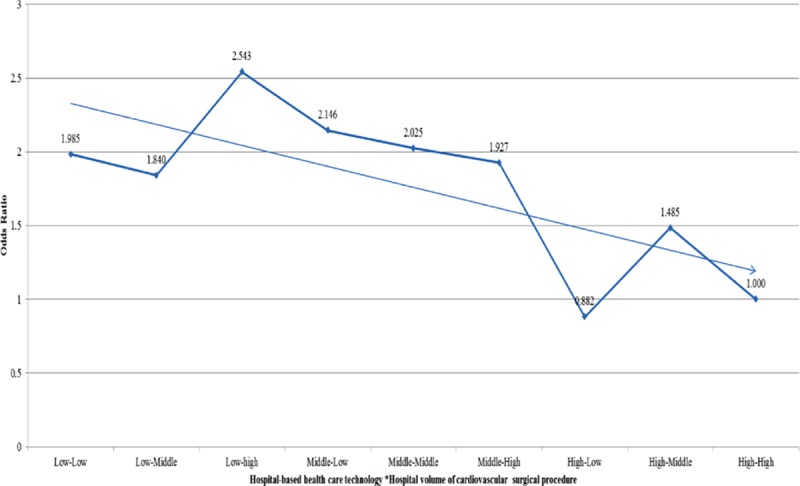
Adjusted effect between hospital-based healthcare technology and 30-day all-cause mortality.

**Figure 2:**
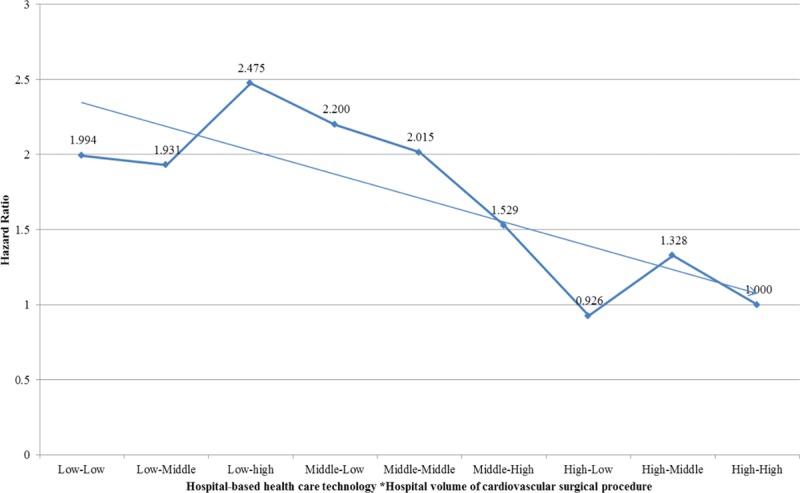
Adjusted effect between hospital-based healthcare technology and 30-day all-cause mortality for percutaneous coronary intervention (PCI) patients.

After adjusting for age, sex, residential region, PCCL, inpatient type, diagnostic code, type of surgery, hospital type, organization type, hospital region, bed, doctor, and magnetic resonance imaging, the odds ratio (OR) of 30-day mortality in low-volume hospitals (model 1) was 1.412-times higher (95% confidence interval [CI]: 1.012–2.013) than in high-volume hospitals (Table 2 , Figure 1). After adjusting for hospital volume of cardiovascular surgical procedures and all other confounders, the OR of 30-day mortality in low healthcare technology hospitals (model 2) was 1.567-times higher (95% CI: 1.069–2.297) than in high healthcare technology hospitals. Model 3 examined the combined effects of hospital-based healthcare technology and hospital volume of cardiovascular surgical procedures as well as all other confounders. The OR of 30-day mortality in low healthcare technology hospitals and low-volume hospitals (low-low) was 1.985 times higher (95% CI: 1.258–3.132) than in high healthcare technology hospitals and high-volume hospitals (high–high). Overall, we found that low healthcare technology hospitals, regardless of volume of cardiovascular surgical procedures, had a higher 30-day mortality rate than high healthcare technology hospitals. Table 3 and Figure 2 show a subgroup analysis of percutaneous coronary intervention patients after adjusting for all confounders, which suggests a trend similar to that seen from an analysis of cardiovascular patients.

**Table 2:**
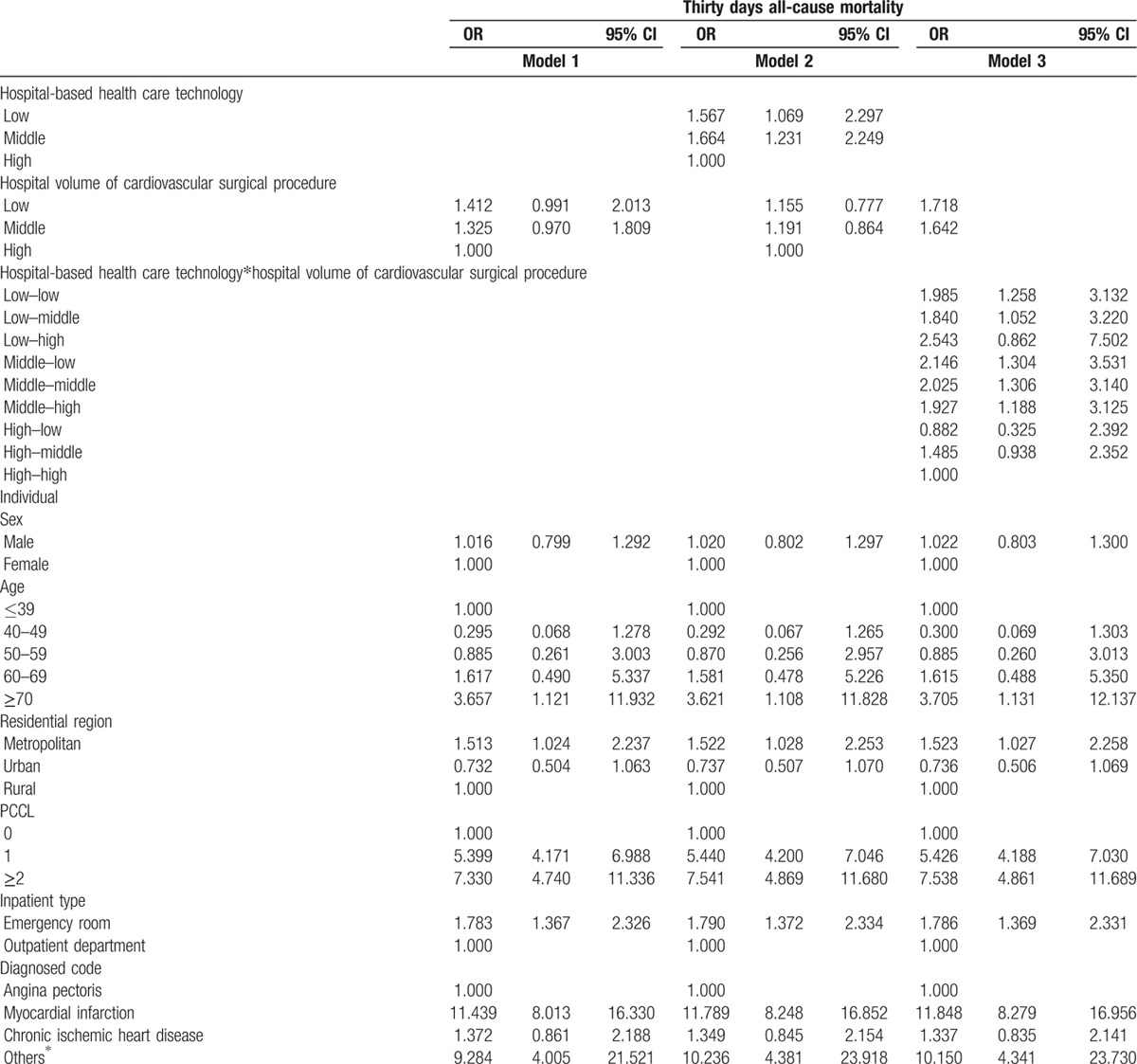
Adjusted effect between hospital-based health care technology and all-cause mortality.

**Table 2 (Continued):**
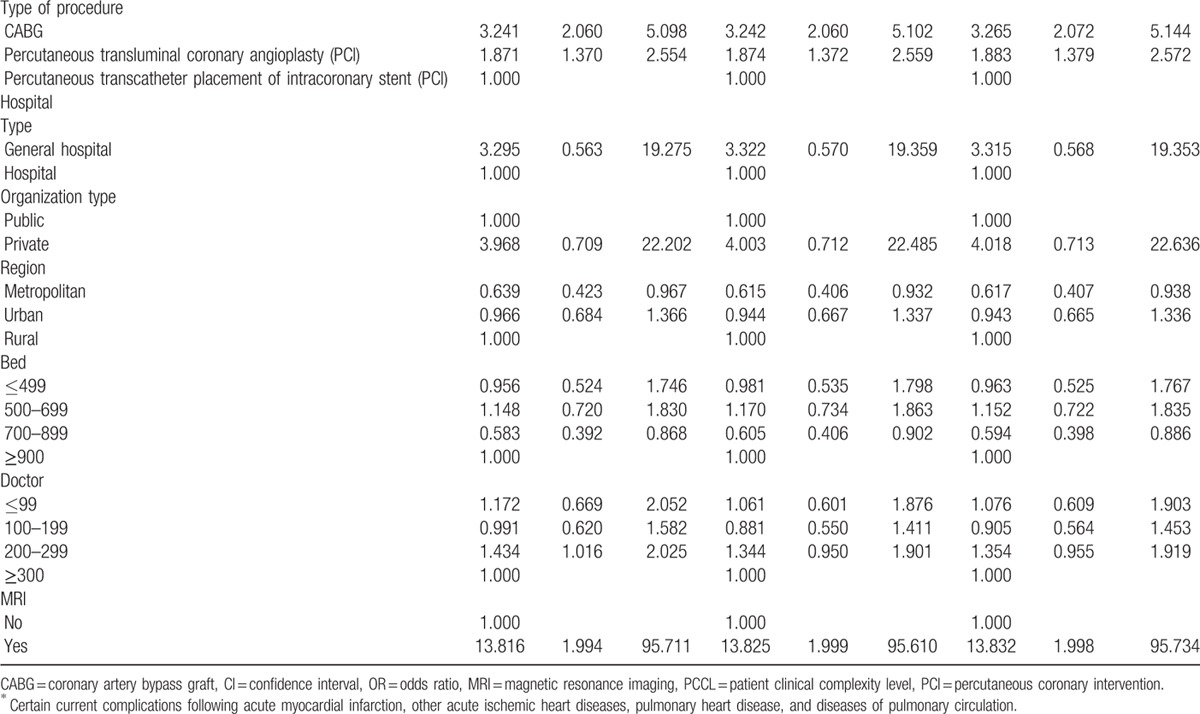
Adjusted effect between hospital-based health care technology and all-cause mortality.

**Table 3:**
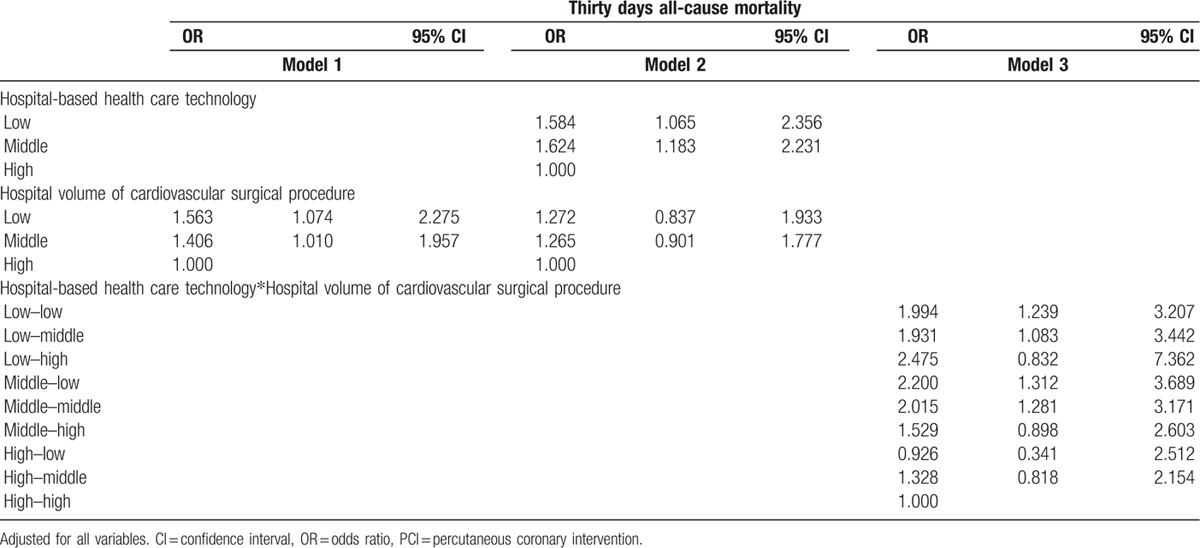
Adjusted effect between Hospital-based health care technology and thirty days all-cause mortality among PCI patients.

## 4 Discussion

In this study, our primary purpose was to investigate whether hospital-based healthcare technology was related to 30-day postoperative mortality rates after adjusting for hospital volume of cardiovascular surgical procedures as well as other covariates in longitudinal models, using nationally representative cohort data from 2002 to 2013 in South Korea. The results of our study provide insightful scientific evidence into the specificity of hospital-based healthcare technology and 30-day mortality in current practice.

The major findings of our study are as follows: hospital-based healthcare technology has a substantial effect on 30-day postoperative mortality among cardiovascular patients (model 2), although hospital volume of cardiovascular surgical procedures is related to 30-day postoperative mortality (model 1). That is, in terms of adjusted effects, hospitals with high healthcare technology are significantly associated with the lowest mortality rates, independent of hospital volume of cardiovascular surgical procedures.

We also found that cardiovascular patients treated in low healthcare technology hospitals, regardless of the extent of hospital volume of cardiovascular surgical procedures, had the highest 30-day mortality rate, followed by cardiovascular patients treated at medium healthcare technology hospitals, whereas cardiovascular patients treated at high healthcare technology hospitals had the lowest 30-day mortality rate regardless of hospital volume of cardiovascular surgical procedures (model 3).

Our results provide considerable evidence indicating that high-volume hospitals have lower mortality rates than low-volume hospitals following complex surgical procedures^[13]^ (model 1). However, despite our observations, opinions regarding the relationship between hospital volume and outcome are controversial.^[6,7]^

There are at least 3 reasons for this controversy. First, many studies of volume and outcome are outdated. Given that healthcare technology has improved and surgical mortality associated with many procedures has fallen considerably, the importance of the volume of procedures may have declined since these studies^[14,15]^ were conducted.^[16]^ Second, although the volume-outcome relationship has been studied extensively, the extent of healthcare technology that may considerably affect patient health outcomes has not yet been explored. Additionally, the relationship between hospital volume and outcome is somewhat of a “black box,” as the mechanisms for this relationship remain unclear.^[17,18]^

The measures used in this study to identify mechanisms contributing to the volume-outcome relationship provided a straightforward assessment of a hospital for an entire system. However, there was a chance that hospital-based healthcare technology may have simply served as a proxy for size or volume. Although measures used to identify these mechanisms correlated with both characteristics, they were far from identical.^[12]^ In fact, a previous study showed that approximately 40% of the 539 hospitals in the highest healthcare technology quintile were medium or smaller-size hospitals.^[12]^

In fact, hospitals vary widely with regard to volume of surgical procedures, teaching status, and health systems, such as the range of services, technologies, resources, and systems of care, which are thought to affect both medical and surgical outcomes for patients with severe disease.^[13,19]^

Nevertheless, high healthcare technology hospitals may be more effective by implementing quality improvement programs such as clinical pathways and protocols that improve the safety of cardiovascular surgical procedures. These improvements may also relate to the teams of healthcare providers that are brought together by specially trained surgeons.^[20]^

Although previous studies have shown that hospital volume is associated with postoperative mortality,^[1,2,5,21]^ there are relatively few studies on the variations in clinical services and technologies as predictors of mortality after cardiovascular surgical procedures. As a result, our study suggests that although a relationship between hospital volume and mortality does exist, at least for cardiovascular surgical procedures (a finding similar to previous studies^[1,2,5,21–23]^), what is more important is that the independent effect of hospital-based healthcare technology may not further improve outcomes in mortality.

Our study has a number of strengths and limitations. The participants in the survey are representative of the overall South Korean cardiovascular inpatient population, as our large and longitudinal cohort sample size allowed the results to be generalized to the adult South Korean population. Nevertheless, several limitations that may have affected our results must be considered in the interpretation of our findings. First, when we selected participants for our study, both ICD coding and cardiovascular surgical patients were considered. However, as hospital-based healthcare technology relied on ICD coding of the principal diagnosis, it was difficult to validate individual ICD codes, particularly given that our data comprised a deidentified database, making it susceptible to errors related to coding. Second, as this was a large and longitudinal nationwide sample, there may have been significant heterogeneity in the care provided both in the field and at receiving hospitals. We cannot comment on which aspects of patient care most affected survival. Third, although unmeasured hospital characteristics including clinical pathways and protocols may have been predictors of mortality, we could not obtain information regarding unmeasured hospital characteristics due to the limited information provided as part of the claim data.

## 5 Conclusion

Our study suggests that increasing overall healthcare technology regardless of the extent of hospital volume should result in lower mortality. Although a variety of factors undoubtedly contribute to the volume–outcome relationship, healthcare technology seems to account for part of the effect observed. In addition to hospital characteristics, such as skill and experience, unmeasured hospital characteristics including clinical pathways and protocols focused on quality are also likely to play an important role. However, further research is required to explore their respective contributions, as evidence for this is unclear.

## References

[1] Dudley RA Johansen KL Brand R Selective referral to high-volume hospitals: estimating potentially avoidable deaths. JAMA 2000;283:1159–66. 10703778 10.1001/jama.283.9.1159

[2] Birkmeyer JD Lucas FL Wennberg DE Potential benefits of regionalizing major surgery in Medicare patients. Eff Clin Pract 1999;2:277–83. 10788026

[3] Chang CM Yin WY Wei CK The combined effects of hospital and surgeon volume on short-term survival after hepatic resection in a population-based study. PLoS One 2014;9:e86444. 24466102 10.1371/journal.pone.0086444 PMC3899267

[4] Joseph B Morton JM Hernandez-Boussard T Relationship between hospital volume, system clinical resources, and mortality in pancreatic resection. J Am Coll Surg 2009;208:520–7. 19476785 10.1016/j.jamcollsurg.2009.01.019

[5] Begg CB Cramer LD Hoskins WJ Impact of hospital volume on operative mortality for major cancer surgery. JAMA 1998;280:1747–51. 9842949 10.1001/jama.280.20.1747

[6] Ihse I The volume-outcome relationship in cancer surgery: a hard sell. Ann Surg 2003;238:777–81. 14631214 10.1097/01.sla.0000098616.19622.af PMC1356159

[7] Christian CK Gustafson ML Betensky RA The leapfrog volume criteria may fall short in identifying high-quality surgical centers. Ann Surg 2003;238:447–55. 14530717 10.1097/01.sla.0000089850.27592.eb PMC1360105

[8] Russell TR Volume standards for high-risk questions: an American College of Surgeons’ view. Surgery 2001;130:423–4. 11562663 10.1067/msy.2001.117137

[9] Hartz AJ Krakauer H Kuhn EM Hospital characteristics and mortality rates. N Engl J Med 1989;321:1720–5. 2594031 10.1056/NEJM198912213212506

[10] Klastorin TDWC A current reappraisal of Berry's hospital typology. Med Care 1982;20:441–9. 7098588 10.1097/00005650-198205000-00001

[11] Department of Veterans Affairs. Veterans Health Administration. Facility infrastructure requirements to perform standard, intermediate, or complex surgical procedures. 2010 Available at: http://www.va.gov/vhapublications/ViewPublication.asp?pub_ID=2227 Accessed October 20, 2013.

[12] McCrum ML Lipsitz SR Berry WR Beyond volume: does hospital complexity matter?: an analysis of inpatient surgical mortality in the United States. Med Care 2014;52:235–42. 24509361 10.1097/MLR.0000000000000077

[13] Birkmeyer JD Siewers AE Finlayson EV Hospital volume and surgical mortality in the United States. N Engl J Med 2002;346:1128–37. 11948273 10.1056/NEJMsa012337

[14] Ghali WA Ash AS Hall RE Statewide quality improvement initiatives and mortality after cardiac surgery. JAMA 1997;277:379–82. 9010169

[15] Katz DJ Stanley JC Zelenock GB Operative mortality rates for intact and ruptured abdominal aortic aneurysms in Michigan: an eleven-year statewide experience. J Vasc Surg 1994;19:804–15. discussion 816–807. 8170034 10.1016/s0741-5214(94)70005-2

[16] Zhao J Booth H Dear K Cardiovascular mortality sex differentials in selected East Asian and Western populations. J Epidemiol Community Health 2016;doi:10.1136/jech-2015–206577. 10.1136/jech-2015-206577 27048151

[17] Ho V Heslin MJ Effect of hospital volume and experience on in-hospital mortality for pancreaticoduodenectomy. Ann Surg 2003;237:509–14. 12677147 10.1097/01.SLA.0000059981.13160.97 PMC1514467

[18] Birkmeyer JD Stukel TA Siewers AE Surgeon volume and operative mortality in the United States. N Engl J Med 2003;349:2117–27. 14645640 10.1056/NEJMsa035205

[19] Allison JJ Kiefe CI Weissman NW Relationship of hospital teaching status with quality of care and mortality for Medicare patients with acute MI. JAMA 2000;284:1256–62. 10979112 10.1001/jama.284.10.1256

[20] Billingsley KG Morris AM Dominitz JA Surgeon and hospital characteristics as predictors of major adverse outcomes following colon cancer surgery – Understanding the volume-outcome relationship. Arch Surg 2007;142:23–31. 17224497 10.1001/archsurg.142.1.23

[21] Nimptsch U Peschke D Mansky T [Minimum Caseload Requirements and In-hospital Mortality: Observational Study using Nationwide Hospital Discharge Data from 2006 to 2013]. Gesundheitswesen 2016;doi:10.1055/s-0042–100731. 10.1055/s-0042-100731 27050140

[22] Shroyer ALW Marshall G Warner BA No continuous relationship between Veterans Affairs hospital coronary artery bypass grafting surgical volume and operative mortality. Ann Thorac Surg 1996;61:17–20. 8561546 10.1016/0003-4975(95)00830-6

[23] Sulzgruber P Sterz F Schober A Progress in the chain of survival and its impact on outcomes of patients admitted to a specialized high-volume cardiac arrest center during the past two decades. Eur Heart J Acute Cardiovasc Care 2015;doi:10.1177/2048872615620904. 10.1177/2048872615620904 26622050

